# Effects of a back school-based intervention on non-specific low back pain in adults: a randomized controlled trial

**DOI:** 10.1186/s12906-023-04061-1

**Published:** 2023-07-10

**Authors:** Pablo Hernandez-Lucas, Raquel Leirós-Rodríguez, Jorge Mota, José L. García-Soidán

**Affiliations:** 1 Faculty of Physiotherapy, University of Vigo, Campus A Xunqueira, 36005 Pontevedra, Spain; 2 SALBIS Research Group, Nursing and Physical Therapy Department, University of Leon, Astorga Ave, 24401 Ponferrada, Spain; 3 Research Center in Physical Activity, Health and Leisure (CIAFEL), Faculty of Sports, FADEUP) and Laboratory for Integrative and Translational Research in Population Health (ITR), University of Porto, Dr. Plácido da Costa St., 91, 4200-450 Porto, Portugal; 4 Faculty of Education and Sport Sciences, University of Vigo, Campus A Xunqueira, 36005 Pontevedra, Spain

**Keywords:** Exercise therapy, Health education, Low back pain, Musculoskeletal pain, Physical therapy modalities, Rehabilitation

## Abstract

**Background:**

Non-specific low back pain is a common condition with significant global prevalence and socio-economic impact. Back School programs, which combine exercise and educational interventions, have been used to address back pain. This study aimed to investigate the effects of a Back School-based intervention on non-specific low back pain in adults. Secondary objectives included evaluating the impact of the program on disability, quality of life, and kinesiophobia.

**Methods:**

A randomized controlled trial was conducted involving 40 participants with non-specific low back pain, who were divided into two groups. The experimental group underwent an 8-week Back School-based program. The program comprised 14 practical sessions focusing on strengthening and flexibility exercises, along with two theoretical sessions covering anatomy and concepts related to a healthy lifestyle. The control group maintained their usual lifestyle. Assessment instruments included the Visual Analogue Scale, Roland Morris disability questionnaire, Short-Form Health Survey-36, and Tampa Scale of Kinesiophobia.

**Results:**

The experimental group showed significant improvements in the Visual Analogue Scale, Roland Morris disability questionnaire, physical components of the Short-Form Health Survey-36, and Tampa Scale of Kinesiophobia. However, there were no significant improvements in the psychosocial components of the Short-Form Health Survey-36. In contrast, the control group did not show significant results in any of the study variables.

**Conclusions:**

The Back School-based program has positive effects on pain, low back disability, physical components of quality of life, and kinesiophobia in adults with non-specific low back pain. However, it does not appear to improve the participants' psychosocial components of quality of life. Healthcare professionals can consider implementing this program to help reduce the significant socio-economic impact of non-specific low back pain worldwide.

**Trial registration:**

NCT05391165 (registered prospectively in ClinicalTrials.gov: 25/05/2022).

## Background

Low back pain is a widespread musculoskeletal condition that is considered the primary cause of years lived with disability [1]. It carries a substantial economic burden, resulting from healthcare expenses, reduced productivity, insurance costs, and sick leave [2–4].

Non-specific low back pain (NSLBP) is the most common type of low back pain and is characterized by the absence of identifiable underlying diseases or anatomical abnormalities [5]. NSLBP typically occurs between the last rib and the iliac crest, although it can also radiate to the gluteus or legs [5]. Patients with chronic NSLBP may experience additional symptoms such as stiffness, muscle weakness, and difficulty with movement [5, 6]. Identifying the primary risk factors associated with NSLBP is crucial to address them effectively and mitigate the significant socio-economic impact it imposes [6]. NSLBP has a multifactorial etiology [7]: involving factors such as sedentary lifestyle [8], obesity [9], smoking [10], inadequate trunk muscle strength [11, 12], poor flexibility [13, 14], psychosocial factors [15, 16] and occupational factors [17, 18].

Prominent clinical practice guidelines recommend a multimodal treatment approach for NSLBP, which combines therapeutic exercise and health education [19–21]. One widely utilized non-pharmacological method for back pain management is the Back School Programme (BSP), which originated in Sweden in 1969 under the direction of physiotherapist Zachrisson Forssell [22]. The BSP consists of a theoretical and practical program designed to impart skills that promote back health [22]. Contemporary NLBP treatment programs adhere to the biopsychosocial model of pain [7]. In line with this model, the updated BSP incorporates recommendations on healthy lifestyles, provides insights into the neuroscience of pain perception, promotes the use of active pain management strategies to reduce fear and catastrophic thinking, clarifies misconceptions about the causes of low back pain, and emphasizes the inherent anatomical resilience of the human spine in its theoretical component [23]. The practical component of the program focuses on teaching patients back strengthening and stretching exercises [24–26].

Scientific evidence supports the beneficial effects of the BSP in individuals with low back pain, including pain reduction [27–35], improved disability [27–37], enhanced quality of life [27–29, 37] and pain prevention [26]. A systematic review published in 2017 raised concerns regarding the low methodological quality of existing studies on the BSP and emphasized the need for further research on new variations of the program [38]. Therefore, the primary objective of this study was to evaluate the impact of an original BSP-based intervention on NLBP in adults. Additionally, the study examined other factors associated with pain, such as disability, quality of life, and kinesiophobia. It was hypothesized that the BSP would yield positive effects, including pain reduction, decreased disability, improved quality of life, and reduced kinesiophobia, in adults with NLBP compared to those who did not participate in the BSP.

## Methods

### Study design

A randomized controlled clinical trial was conducted, wherein scores on dependent variables were compared before and after the intervention in both the experimental group (EG), consisting of individuals who participated in the BSP, and the control group (CG), comprising individuals who indicated that they would not modify their lifestyle during the study. The experimental procedures adhered to the CONSORT guidelines. The study protocol received approval from the University of León Research Ethics Committee (code: ULE-035–2022) and was registered on ClinicalTrials.gov with the ID: NCT05391165 (registered prospectively on 25/05/2022). All participants provided informed consent to participate, following the principles outlined in the Declaration of Helsinki (2013 version).

### Participants

A total of forty volunteers meeting the following inclusion criteria participated in the study: (i) aged between 18 and 65 years old, and (ii) experiencing non-specific low back pain for a minimum of three months, with pain intensity ranging from 30 to 70 on the Visual Analogue Scale (VAS). The study also applied the following exclusion criteria: (i) a history of cancer, spinal infection, rheumatologic diseases, spine fracture, red flag signs (such as significant and unexplained weight loss exceeding 10% of total body weight within the past six months, and presence of fever), psychological disorders, and previous spine surgery, radiculopathy, anatomical or congenital abnormalities; (ii) attendance of fewer than two BSP sessions; (iii) inability to attend the measurement sessions. The participants were randomly allocated into two groups with a 1:1 ratio, and the assignment was concealed using sealed opaque envelopes.

### Intervention

The intervention consisted of a novel and original programme based on the BSP, which follows the recommendations of the biopsychosocial model of chronic pain [7]. The intervention was carried out in the physiotherapy area of a sports centre. The duration of the intervention was eight weeks with a frequency of two sessions per week, making a total of 16 sessions of 45 min duration each. Out of all the sessions, 14 had a practical focus and the other two had a theoretical focus. A summary of the intervention and procedure carried out in this study is shown in Table 1. All sessions were given by a registered physiotherapist and were conducted face-to-face and in groups of maximum 10 participants.

**Table 1:** Summary of the intervention and procedure

Session number	Session type	Name	Main objective of the session
2	Theory	Anatomy and NLBP risk factors	Learn the basics of anatomy, biomechanics, and clarification of erroneous beliefs regarding the causes or origin of NLPB
3–5	Practice	Exercises without implements	Do and learn strength and flexibility exercises without implements
6	Theory	Psychosocial NLBP risk factors	Learn about psychosocial NLBP factors and stress management techniques
7–8	Practice	Exercises without implements	Do and learn strength and flexibility exercises without implements
9–11	Practice	Exercises with elastic band	Do and learn strength and flexibility exercises with a light resistance band
12–14	Practice	Exercises with toning ball	Do and learn strength and flexibility exercises with the 0.5 kg toning ball
15–17	Practice	Exercises with dumbbell	Do and learn strength and flexibility exercises with the 1 kg dumbbell

(a) Theoretical sessions: The first session aimed to explain basic concepts of anatomy and biomechanics of the spine with the help of anatomical models and videos, and to inform about erroneous catastrophic beliefs about the causes and origin of NLBP. The second session aimed to easily explain the main psychosocial factors that can influence the perception of pain, such as emotions or previous experiences of pain, by means of pictures and examples.

(b) Practical sessions: The practical sessions followed a structured format, consisting of four parts: doubts, warm-up, main part, and cool-down. The first part, lasting approximately three minutes, allowed participants to ask questions and review the fundamental principles of each exercise. In sessions where no doubts were raised, the physiotherapist utilized this time to inquire about the topics covered in the theoretical classes, aiming to reinforce knowledge integration between theory and practice.

The warm-up phase lasted seven minutes and involved joint mobility exercises. The main part of the session spanned 30 min and included a variety of exercises such as squats, isometric abdominal exercises, alternative arm/leg extensions, lateral trunk/leg raises, arm/leg raises, shoulder bridges, abdominal exercises with controlled exhalation, oblique abdominal exercises with controlled exhalation, rolling onto the back, abdominal exercises raising opposite arm/leg with controlled exhalation, rolling from one sacroiliac joint to the other, abdominal exercises with controlled exhalation and leg raise to 45º, alternate leg lifts with spine elongation, alternate arm/leg lifts with spine elongation, and simultaneous arm/leg lifts with spine elongation. Throughout this phase, exercises aimed to activate the trunk stabilizing muscles were interspersed with active breaks consisting of gentle stretching and joint mobility exercises.

For the first five practical classes, exercises were performed without any equipment. In the subsequent three sessions, a light resistance band was introduced as an implement. Sessions 12, 13, and 14 incorporated the use of a half-kilogram toning ball, while the last three classes utilized a one-kilogram dumbbell for resistance.

The cool-down phase lasted five minutes and focused on flexibility, breathing exercises, and relaxation techniques. Practical sessions were conducted in groups, with a maximum of 10 participants.

### Variables analysed

Two assessment sessions were conducted at the start and conclusion of the intervention to gather sociodemographic and anthropometric information. Participants' age, sex, weight (measured with a Tanita™ b303 scale, Tokyo, Japan), and height (measured with a standardized Seca™ 709 height rod, Hamburg, Germany) were recorded during these sessions.

Pain intensity was assessed using the Visual Analogue Scale (VAS), a widely used tool for measuring pain. Participants were asked to indicate their perceived pain intensity, typically over the past 24 h, by marking a point along a 100 mm horizontal line. The left edge of the line represents the absence of pain, while the right edge represents the highest intensity of pain [39].

Disability was assessed using the Roland Morris Disability Questionnaire (RMDQ), a 24-item patient-reported outcome measure that evaluates pain-related disability associated with low back pain. Each item is scored as 0 if left blank or 1 if endorsed, resulting in a total RMDQ score ranging from 0 to 24. Higher scores indicate higher levels of pain-related disability [40]. In this study, the Spanish validated version of the RMDQ was utilized [41].

The quality of life was assessed using the Spanish version of the 36-Item Short-Form Health Survey (SF-36) [42]. This survey comprises eight dimensions that cover various aspects of health-related quality of life: physical functioning, role limitations due to physical health problems, bodily pain, general health perceptions, vitality, social functioning, role limitations due to emotional problems, and general mental health. These eight dimensions can be summarized into two main components: the physical component summary (PCS) and the psychosocial component summary (MCS) of the SF-36 [43]. Scores on each dimension and the summary components range from zero (indicating the worst health status) to 100 (representing the best health status) [43].

The degree of kinesiophobia was measured using the Spanish version of the Tampa Scale of Kinesiophobia (TSK-11) [44]. This scale comprises 11 questions, each with four possible answers. The total score on the scale ranges from 11 to 44, with a lower score indicating no kinesiophobia and a higher score indicating severe kinesiophobia [44, 45].

### Statistical analysis

The statistical analysis was conducted according to the intention-to-treat principle, where all participants, including those who withdrew from treatment or had poor compliance, were included in the analysis based on their assigned group. Missing values were estimated using Multiple Imputation by linear regression for continuous variables. Descriptive statistics such as mean, standard deviation, median, and interquartile range were used to summarize the data. The normal distribution of the groups was assessed using the Shapiro–Wilk test, and homogeneity was confirmed using Levene's test. To evaluate the effect of the intervention, the non-parametric Mann–Whitney test was used since the difference between post-intervention and baseline scores did not follow a normal distribution. Effect sizes were interpreted using Hedges' g based on Cohen's guidelines [46], where values of 0 to 0.2 were considered very small, 0.2 to 0.5 as small, 0.5 to 0.8 as moderate, and 0.8 or higher as strong.

The significance level was set at *p* < 0.05, and all statistical analyses were performed using Stata 16.0 for MacOS® software (Stata Corporation, College Station, TX, USA).

## Results

**Fig. 1:**
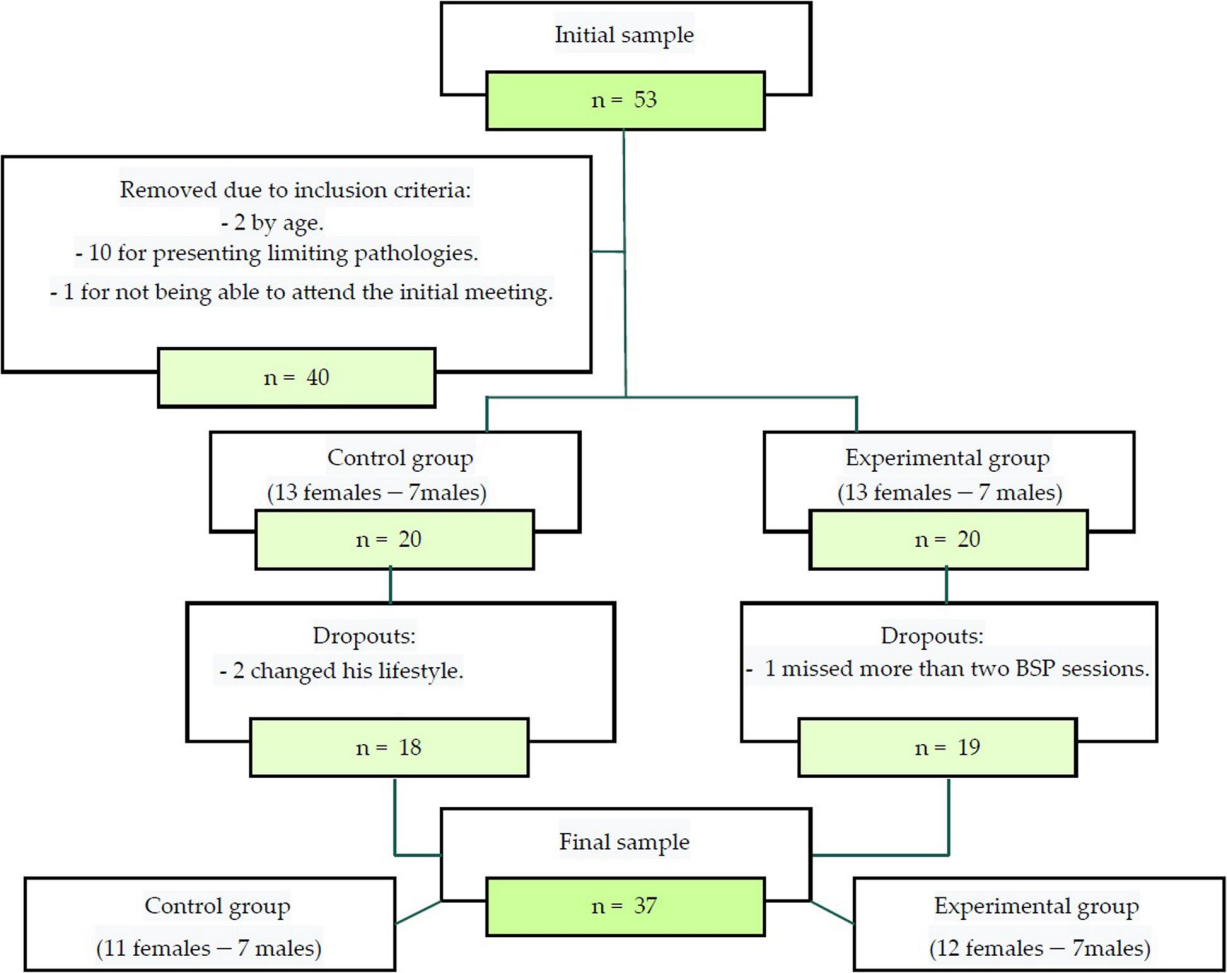
Sample selection flowchart

The sample size for the study initially included 40 participants who met the inclusion and exclusion criteria. However, three participants dropped out during the course of the study, resulting in a final sample size of 37 participants. Among the final participants, there were 23 women and 14 men (Fig. 1). A post-hoc power analysis (1—β err prob) was conducted using the final sample size of 37 participants, yielding a power of 0.9 for a significance level of *p* < 0.05 [27]. This indicates that the study had a high probability of detecting statistically significant effects if they existed. It is worth noting that none of the participants reported any adverse effects during the study. Baseline values of the study variables are presented in Table 2.

**Table 2:** Baseline of the studied variables

Variable	All (*n* = 40)			CG (*n* = 20)			EG (*n* = 20)		
	Mean ± SD	Median	IQR	Mean ± SD	Median	IQR	Mean ± SD	Median	IQR
Age (Years)	50.5 ± 7.8	51	7	49.9 ± 8.6	50.5	6	51.1 ± 7.1	51	7
Weight (Kg)	62.6 ± 9.4	59	15	63.3 ± 9	60.5	17.5	61.9 ± 10	58.1	12.4
Height (cm)	164.7 ± 10.4	160	14	165 ± 9	162.5	17	164.4 ± 121	159.5	14
BMI (Kg/m^2^)	23 ± 1.1	23	1.5	23.1 ± 1.4	23.2	1.3	22.8 ± 0.8	22.8	1.4
VAS (mm)	53.4 ± 9.3	55	10	53.3 ± 9.8	55	10	53.6 ± 9.1	52.5	10
RMDQ	6 ± 4.1	5	6	5.4 ± 3.9	4	7	6.6 ± 4.3	6	5
fSF-36	41.2 ± 8.3	41.6	10.6	43.1 ± 7.5	43.1	9.9	39.4 ± 8.9	37.5	11.9
pSF-36	45.2 ± 8.7	45.5	12	44.3 ± 7.7	44.5	13.5	46.1 ± 9.8	46.3	10
TSK-11	25.9 ± 5.4	25	6	26.7 ± 5	25.5	7	25.2 ± 5.7	24.5	7

*CG* Control group, *EG* Experimental group, *SD* Standard deviation, *IQR* Interquartile Range, *BMI* Body mass index, *VAS* Visual Analogue Scale, *RMDQ* Roland Morris disability questionnaire, *fSF-36* physical components Short-Form Health Survey-36, *pSF-36* psychosocial components Short-Form Health Survey-36, *TSK-11* Tampa Scale of Kinesiophobia

In the analysis per intention to treat of the outcomes, significant differences in change between both groups were found on VAS (*U* = 305.5; *p* = 0.004; *g* = -0.97), RMDQ (*U* = 357; *p* < 0.001; *g* = -2.257), fSF-36 (*U* = 331;* p* < 0.001; *g* = -1.96) and TSK-11 (*U* = 348; *p* < 0.001; *g* = -1.64) while no significant interaction was found on pSF-36 (*U* = 211; *p* = 0.779; *g* = -0.09) (Table 3).

**Table 3:** Inferential statistics per intention to treat

Variable	Group (N)	Pre-test		Post-test		*p* value
		Mean ± SD	Median (IQR)	Mean ± SD	Median (IQR)	
VAS (mm)	CG (20)	53.3 ± 9.8	55 (10)	38.9 ± 9.8	40 (19)	*p* = 0.004
	EG (20)	53.6 ± 9.1	52.5 (10)	26.1 ± 10.5	25 (10)	
RMDQ	CG (20)	5.4 ± 3.9	4 (7)	4.8 ± 2.9	4.8 (5)	*p* < 0.001
	EG (20)	6.6 ± 4.3	6 (5)	2.8 ± 2.6	2 (3)	
fSF-36	CG (20)	43.1 ± 7.5	43.1 (9.9)	43.9 ± 6.7	44.4 (7.4)	*p* < 0.001
	EG (20)	39.4 ± 8.9	37.5 (11.9)	46.7 ± 6.2	47.3 (8.2)	
pSF-36	CG (20)	44.3 ± 7.7	44.5 (13.5)	47.4 ± 8.3	46.6 (13.4)	*p* = 0.779
	EG (20)	46.1 ± 9.8	46.3 (10)	49.9 ± 8.2	54.5 (13)	
TSK-11	CG (20)	26.7 ± 5	25.5 (7)	26.1 ± 4.7	25 (8)	*p* < 0.001
	EG (20)	25.2 ± 5.7	24.5 (7)	19.6 ± 6.3	19 (9)	

*N* Sample, *SD* Standard deviation, *IQR* Interquartile Range, *CG* Control group, *EG* Experimental group, *VAS* Visual Analogue Scale, *RMDQ* Roland Morris disability questionnaire, *fSF-36* physical Short-Form Health Survey-36, *pSF-36* psychosocial Short-Form Health Survey-36, *TSK-11* Tampa Scale of Kinesiophobia

The per-protocol analysis was restricted to 37 patients: 19 in the EG and 18 in the CG. These patients were excluded if they missed more than two BSP sessions in the EG or if they changed their lifestyle in the CG. The results of the per-protocol analysis (Table 4) were similar to the results of the intention-to-treat analysis (Table 3). Both analyses showed significant differences in VAS, RMDQ, SF-36, and TSK-11, while neither showed significant differences in pSF-36.

**Table 4:** Inferential statistics per protocol

Variable	Group (N)	Pre-test		Post-test		*p* value
		Mean ± SD	Median (IQR)	Mean ± SD	Median (IQR)	
VAS (mm)	CG (18)	53.6 ± 8.9	55 (10)	38.2 ± 10	40 (16)	*p* = 0.003
	EG (19)	53.8 ± 9.3	55 (10)	25.3 ± 10.2	25 (10)	
RMDQ	CG (18)	5.2 ± 3.7	4 (7)	4.7 ± 3	4 (5)	*p* < 0.001
	EG (19)	6.5 ± 4.4	5 (5)	2.8 ± 2.6	2 (3)	
fSF-36	CG (18)	42.5 ± 7.3	42.5 (10)	43.4 ± 6.7	44.6 (7.8)	*p* < 0.001
	EG (19)	39.6 ± 9.1	37.7 (12.5)	46.5 ± 6.3	46.2 (8.3)	
pSF-36	CG (18)	44.8 ± 7.7	44.5 (12.7)	47.7 ± 8.3	46.6 (13.2)	*p* = 0.730
	EG (19)	46.2 ± 10.1	46.6 (10.6)	49.8 ± 8.5	54.7 (13.1)	
TSK-11	CG (18)	26.1 ± 4.8	25 (6)	25.6 ± 4.6	25 (8)	*p* < 0.001
	EG (19)	25.1 ± 5.9	24 (8)	19.3 ± 6.3	19 (8)	

*N *sample, *SD* Standard deviation, *IQR* Interquartile Range, *CG* Control group, *EG* Experimental group, *VAS* Visual Analogue Scale, *RMDQ* Roland Morris disability questionnaire, *fSF-36* Physical Short-Form Health Survey-36, *pSF-36* Psychosocial Short-Form Health Survey-36, *TSK-11* Tampa Scale of Kinesiophobia

## Discussion

The aim of this research was to determine the effects of a BSP-based intervention for the treatment of patients with NLBP in an adult population. The results of the study suggest that the effects are positive, including pain reduction, improvement of disability, improvement in the physical components of quality of life, and reduction in kinesiophobia.

Participants in the study demonstrated minimal clinically important differences in pain intensity [47]. This can be attributed to the fact that risk factors influencing NLBP can be addressed through a biopsychosocial approach [48], which aligns with the BSP intervention based on the biopsychosocial model of pain [7]. Consistent with these findings, a systematic review published in 2022 concluded that integrating psychosocial interventions and exercise is more effective in the short-term treatment of NLBP compared to standard medical care. Furthermore, inadequate trunk muscle strength and flexibility have been associated with increased NLBP [11, 12, 14]. Previous studies using the BSP and similar programmes have demonstrated improvements in strength and flexibility [26, 27, 49].

Although the clinically important difference in pain intensity is minimal, it is worth noting that other interventions [27, 49], required a higher frequency of sessions (three sessions per week) to achieve these changes. This distinction can be useful for optimizing the dosage of such programmes to increase their efficacy. Additionally, the observed improvements in disability have been clinically significant according to the definition by Ostelo et al. [47]. These results are consistent with the strong association between pain and disability, given the close relationship between physical and psychosocial factors [50]. Other studies have also reported significant improvements in disability [27, 28, 30–37, 49], with some noting that these improvements were sustained for several weeks after the programme [31, 34, 36] or even up to two years later [32].

Previous studies have provided evidence for the positive effects of the BSP intervention on the overall quality of life [27–29, 37]. However, in our study, no significant changes were observed in the psychosocial component of quality of life. It is important to note that the sample size for this particular intervention was smaller compared to the other studies. Nonetheless, even though the results did not reach statistical significance, there was a positive trend observed. This finding is clinically meaningful, as it aligns with the strong relationship between pain, disability, kinesiophobia, and quality of life [51].

A previous study examining the effects of BSP on kinesiophobia also reported positive outcomes [49]. However, it is important to note that the improvements in kinesiophobia observed in our intervention were 62.5% higher than those reported in the study by Martijn et al. [49]. This finding holds significant importance since kinesiophobia is known to be a negative predictor of favourable outcomes in NLBP [52]. Furthermore, individuals with NLBP and higher levels of kinesiophobia tend to experience more pain and have lower quality of life [53]. This is closely linked to their reduced physical activity levels, which is a risk factor for the chronicity of NLBP [8, 53]. The International Association for the Study of Pain recognizes the relationship between fear, pain, and knowledge, as they emphasize that pain is not only a physical sensation but also an emotional experience influenced by other emotions such as anxiety and fear of the unknown [54]. Hence, the biopsychosocial approach aligns with the current paradigm in NLBP treatment [7]. It is also noteworthy that disability is related to kinesiophobia [55], thus confirming the benefits observed in both variables in our study.

Based on the results obtained in this study, it appears that BSP may be beneficial in the treatment of NLBP. This aligns with the findings of a review of Clinical Practice Guidelines, which emphasizes the importance of exercise therapy and health education for favorable outcomes in individuals with NLBP [19].

However, there are several limitations to consider in this study. First, due to the study design, neither the participants nor the evaluators were blinded, which may introduce bias in the results. Additionally, certain physiological factors such as menopause and occupational factors like the type of work or number of hours were not taken into account in the data analysis, potentially influencing the outcomes. It is important to note that our study did not include a post-intervention follow-up, limiting the assessment of long-term effects. Lastly, the limited number of participants hindered the stratification of results by age and gender.

For future research, it would be valuable to incorporate post-intervention follow-ups to evaluate the sustainability of the effects over time. Additionally, larger sample sizes would enable stratified analysis to explore potential age and gender differences in response to the intervention. These improvements would contribute to a more comprehensive understanding of the effectiveness of BSP in treating NLBP.

## Conclusions

The BSP-based intervention had beneficial effects on pain in patients with NLBP. In addition, this programme improved disability, physical components of quality of life and kinesiophobia in patients with NLBP. However, the psychosocial components of quality of life did not change after participating in the BSP. This programme could be easily implemented in hospitals, primary care centres and physiotherapy clinics with the aim of reducing the severe socio-economic impact caused by NLBP worldwide.

## Data Availability

The datasets used and/or analysed during the current study are available from the corresponding author on reasonable request.

## Abbreviations

NSLBP: Non-specific low back pain
BSP: Back School Program
VAS: Visual Analogue Scale
RMDQ: Roland Morris disability questionnaire
fSF-36: Physical components Short-Form Health Survey-36
pSF-36: Psychosocial components Short-Form Health Survey-36
TSK-11: Tampa Scale of Kinesiophobia
